# Coping with the Blues: Simple and Photo‐Stable Dye for Long‐Term Live‐Cell Imaging

**DOI:** 10.1002/chem.202501360

**Published:** 2025-10-17

**Authors:** Justine V. Schwarte, Christophe Lamy, Salves Cornelis, Ora Hazak, Katharina M. Fromm

**Affiliations:** ^1^ Department of Chemistry University of Fribourg Chemin du Musée 9 Fribourg 1700 Switzerland; ^2^ Department of Biology University of Fribourg Chemin du Musée 5 Fribourg 1700 Switzerland; ^3^ Section of Medicine University of Fribourg Chemin du Musée 8 Fribourg 1700 Switzerland; ^4^ Institute of Plant Biology and Biotechnology University of Münster Schlossplatz 4 48149 Münster Germany

**Keywords:** fluorescent probes, imaging agents, light induced configuration change, photobleaching stability, photochemistry

## Abstract

Dyes for long‐term imaging of live cells are rare, especially in the blue region, as they need to be nontoxic and photo‐stable. New simple pyrene‐derived dyes have been synthesized and fully characterized, showing good cellular uptake, intense emission, and excellent stability. Their unusual stability secret lies in their flexible conformation, evidenced by the solid‐state structures of coordination compounds obtained from the dyes and by ^1^H‐NMR solution studies. Results show that under irradiation, the pyrene‐based dye undergoes intersystem crossing (ISC) that leads to a configurational switching occurring at time scales that are faster than the formation of possible photobleaching products. Irradiation of these dyes over 24 hours shows great photostability that was evaluated in different solvents. The possible mechanism of the stability of these dyes is discussed here. Being nontoxic and emitting at only a specific wavelength, these compounds are perfect for staining the cytosol of live cells and long‐term imaging in parallel with other dyes of different colors.

## Graphical abstract

PyPe is a fluorescent emitter with low photobleaching thanks to its vinyl moiety serving as a radiationless relaxation group. This favors the configuration flip between E‐ and Z‐isomers at excited states and promotes photostability. Then, photodimers form in various kinetics and proportions depending on temperature and complexation. Low toxicity makes it a blue photostable dye, readily taken up by cells, and suitable for bioimaging experiments.

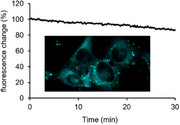

## 1 Introduction

The observation of living cells to gain a better understanding of their functioning requires dyes that are photostable over prolonged periods of recording under the conditions of laser irradiation used in laser scanning confocal microscopy (*LSCM*).^[^ ^1^, ^2^ ^]^ Moreover, different cell parts need to be stained with different dyes that absorb at unique wavelengths in order not to disturb the readouts.^[^ ^3^ ^]^ Most current fluorophores that are used in such applications, for example AlexaFluor 405, 488,^[^ ^4^ ^]^ Cascade Blue,^[^ ^5^ ^]^ Atto 488,^[^ ^4^ ^]^ Cyanine dyes^[^ ^6^ ^]^ typically suffer from bleaching and/or chemical degradation over a very short time upon repeated irradiation, resulting in the loss of emission intensity, not being detectable anymore after several minutes.^[^ ^7^, ^8^, ^9^, ^10^, ^11^ ^]^ Improved, more stable small dye molecules are complex and require heteroatoms, complex functional groups, and a complicated synthesis.^[^ ^12^ ^]^ Most of them emit in the red, orange, or green range of visible light.^[^ ^13^ ^]^ To avoid photobleaching, scientists have also turned to quantum dots based on nanoparticles of 10–20 nm^[^ ^14^ ^]^ with tunable fluorescence, but their preparation requires expensive processes such as coating and functionalization.^[^ ^15^ ^]^ Therefore, the synthesis and investigation of nonphotobleachable dyes for cell staining is still a challenge. Thus, fast photobleaching is an enormous limitation for long‐term exposure experiments, and preventative measures against photobleaching must be taken to improve the stability of the dyes. A well‐known mechanism of photobleaching is the process that brings an electron of a molecule upon irradiation with the light from the singlet excited state (S_1_*) to the forbidden excited triplet‐states (T_n_*) through intersystem crossing (*ISC*). At the triplet state, the chromophore/dye is very reactive and may interact with another dye molecule or with the environment, for example solvent or oxygen molecules, resulting in photobleached dye product(s).^[^ ^16^, ^17^, ^18^ ^]^ Preventive strategies for stabilizing the dyes are not yet fully understood, as many factors could stabilize the dye and stop it from photobleaching.^[^ ^19^, ^20^ ^]^

Here we demonstrate a simple pyrenyl‐based dye that has a remarkable photo‐stability in the conditions of a bio‐imaging experiment, due to a vinylene‐functional group that is incorporated into a chromophore pyrenyl‐pyridine (**PyPe**).

## 2 Results and Discussion

### 2.1 Isomerization

**Figure 1:**
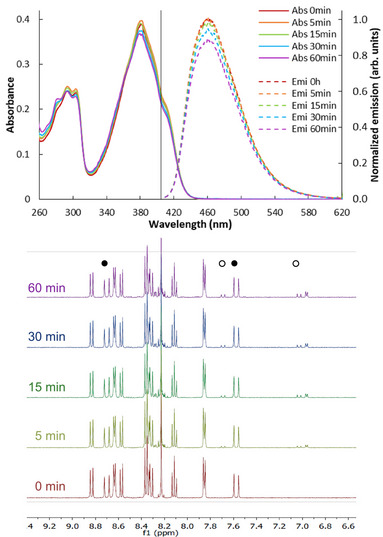
Top: UV‐Vis (full) and fluorescence (dashed) spectra obtained after irradiation of **PyPe** for the mentioned time with 400nm light (vertical line), Hg‐arc lamp, 400±25 nm). Bottom: ^1^H‐NMR experiments reveal configuration changes from E‐ to Z‐ (new signals at 7.03 and 7.69 ppm). Annotated peaks correspond to the vinyl bond of ● E and Z isomers.

1‐(pyren‐1‐yl)‐2‐(pyridin‐4‐yl)ethan‐1‐ol (1, *E‐* **PyPe**) and its *d^10^*‐metal coordination compounds [MPyPe_2_I_2_] (with M corresponding to Zn, Cd, and Hg, respectively, complexes will be named **ZnPyPe**, **CdPyPe**, and **HgPyPe**) were synthesized and characterized according to previous work.^[^ ^21^ ^]^ PyPe absorption and emission properties were measured in different solvents (Figure 1, red curve, and Figure S1 and Table S1). In water, the scattering shown by UV spectra demonstrated the poor solubility of **PyPe**, but organic solvents resulted in solutions with molar absorption coefficient ranging from 6557 to 13 917 L*mol^−1^*cm^−1^ (acetonitrile vs. DMSO), and quantum yield from 0.51 to 0.92 (THF vs. acetonitrile). Absorption maxima were around 370 nm, and emission was around 452 nm. Blue emitter dyes (emission between 450 and 500 nm) are rare, representing only 8% of available dyes versus more than 60% for green emitters (500 to 600 nm),^[^ ^13^ ^]^ and the properties of *E‐* **PyPe** were therefore investigated further.

In an irradiation experiment, *E‐* **PyPe** was exposed to excitation by 400 nm light obtained from an Hg‐arc lamp and filter (Figure S2). The irradiation was monitored by UV‐Vis and fluorescence spectrometry (Figure 1), which showed no significant changes over 1 hour in absorption intensity and position of the bands. The low decrease of intensity over 1 h (9 % loss) is considered negligible at bio‐imaging timescale, as cells are irradiated only for a limited time to prepare the pictures under a confocal microscope.

**Figure 2:**
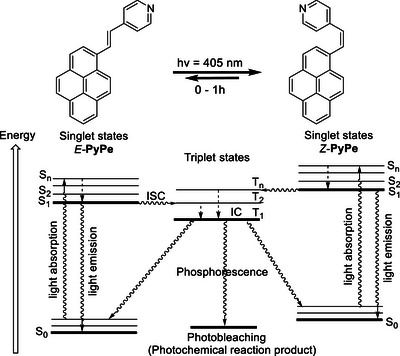
Jablonski diagram of the photobleaching resistance of **PyPe** shows that at the triplet excited state (T_1_) **PyPe** switches configuration from E‐ to Z‐isomer. Whereas the relaxation from T_1_ to S_0_ or S’_0_ results generally in phosphorescence emission, the presence of a stilbene motif in **PyPe** structure induces a faster dissipation of the energy, leading to fluorescence.

This loss of intensity has to be linked to the apparition of photoproducts, identified from the NMR spectra. During the first hour, the apparition of low‐intense new chemical shifts at 6.9, 7.0, and 7.7 ppm is observed. These are characteristic of the *Z‐*isomer, correlated with the decrease of the *E‐*signals at 7.6 and 8.7 ppm, and evidence the isomerization of the double bond of **PyPe** molecules during light irradiation (Figure 2).

Similar to stilbene, which displays a short half‐life (about 1 ns),^[^ ^22^ ^]^ the *vinylene*‐linker serves as a radiationless relaxation functional group ^[^ ^23^ ^]^ that favors the configuration flip at excited states. By taking into account that at the excited T_n_* states, π‐orbitals of the *vinylene*‐bond are oriented perpendicularly to each other,^[^ ^24^ ^]^ σ‐bond rotation along *vinylene* bond can occur on ± 90° on equal directions, leading to the photodynamic equilibrium of *E/Z*‐**PyPe**.^[^ ^25^ ^]^ Indeed, the *E*/*Z*‐isomerization rates of stilbene are much faster at the relative time scale *k*_isc_ of ISC than the possible rates of photobleaching products formation *k*_b_. Presumably, the photobleaching may also not occur in *E‐* **PyPe** due to *pyrenyl*‐chromophore hindrance at the excited state (*S*_n_*, *T*_n_*). Thus, the efficient sort of “wiggling” of the *pyrenyl*‐chromophore through the *vinylene*‐linker group prevents close proximity and chemical interactions at the excited states (*T*_n_*) with another molecule of **PyPe** or solvent, or oxygen molecules. This concludes that the dyes based on the vinylene moiety should resist long, high‐power, and intense light irradiation in experiments such as *LSCM* for bioimaging and *STED* experiments. The backward photoreaction from *Z* to *E* also takes place but with less efficiency,^[^ ^26^ ^]^ explaining the regular and slow increase of *Z‐* **PyPe**.

**Figure 3:**
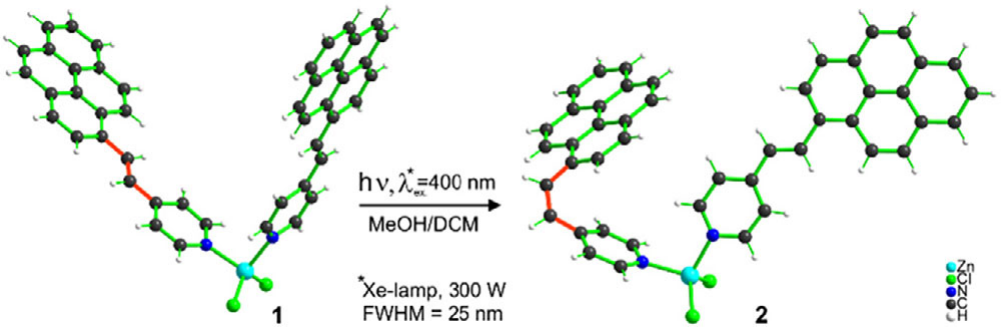
Crystal structures of the complexes with **PyPe** and Zn^2+^ before irradiation [Zn(E‐**PyPe**)_2_Cl_2_]∙2DCM (**1**) and after irradiation with 400 nm light 300 W, Hg arc lamp (FWHM = 25 nm) [Zn(E‐**PyPe**)(Z‐**PyPe**)Cl_2_] (**2**).

An additional proof of the efficient isomerization of **PyPe** can be observed through the irradiation at 400 nm of the coordination complex of *E*‐**PyPe** with zinc (II) chloride, [Zn(*E*‐**PyPe**)_2_Cl_2_] ∙2DCM (**1**) (Figure 3), where two ligands of **PyPe** remain in *E‐*configuration after coordination to a Zn^2+^ ion. However, after exposure to 400 nm light for 24 hours, we were able to isolate a complex in which one of the ligands had changed its configuration to *Z*, while the other remains in *E*, resulting in [Zn(*E*‐**PyPe**)(*Z*‐**PyPe**)Cl_2_] (**2**) (Figure 3). This conformational flip induced changes for some parameters, described in Table 1. Besides the angle increasing between pyridine and pyrene (47.6° instead of 2.6°), due to the destabilization of *Z‐* **PyPe** (the overlapping of two protons from pyridine and pyrene moiety prevents the conjugated system to be flat (Figure 3)), there is a trend for a larger N─Zn─N angle for complex **2** by ca. 4° compared to the complex with all E.

**Table 1:** Crystal structure parameters of complexes **1** and **2**.

	1	2	
	*E*‐isomer	*E*‐isomer	*Z*‐isomer
M─N distance (Å)	2.0363 (41)	2.0426 (93)	2.0353 (94)
M─Cl distance (Å)	2.2102 (41)	2.1957 (29) and 2.2387 (31)	
N1─M─N2 angle	99.99 (23)°	104.63 (41)°	
Torsion angle between pyrene / pyridine planes	2.64°	3.61°	47.64°

However, with **PyPe** in the *Z‐*conformation, the ligand cannot adopt a fully planar structure due to steric hindrance between the protons of the pyridine and the pyrene (in α of the vinyl bond). Instead, an angle of 48.4° between the pyrene and the pyridine planes is observed in the solid state structure (3.6° for the *E‐*isomer). This partial loss of conjugation explains why the *Z‐*isomer does not have the same photochemical properties as the *E‐*, and therefore the slight decrease in absorbance and emission at the beginning of the irradiation.

### 2.2 Dimerization

**Figure 4:**
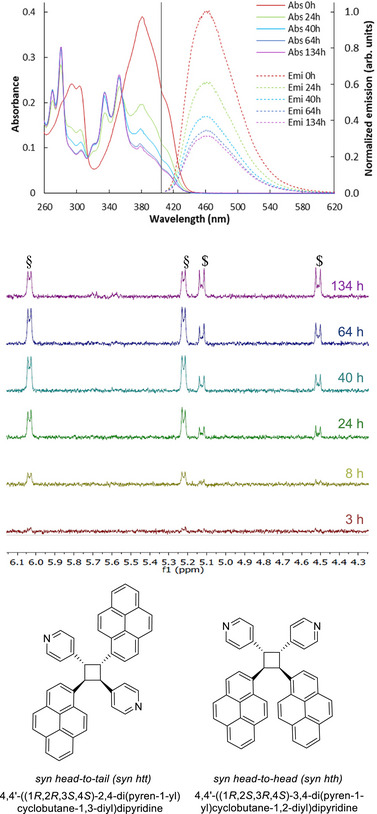
a) Absorbance and emission spectra after long irradiation time b) New ^1^H NMR signals c) Potential isomers resulting from a [2 + 2] cycloaddition. Annotated peaks correspond to the cyclobutyl protons of (§) syn htt and ($) syn hth isomers.

Longer irradiation led to the continuous decrease of signals for the E‐isomer on NMR spectra, while Z‐signals reached a plateau after 1 hour (around 9–13% of Z‐PyPe). In parallel, low‐intensity signals appear after 3 hours (set of protons at 5.2 and 6.0 ppm) and 8 hours (set of protons at 4.5 and 5.1 ppm) (Figure 4b, full NMR spectra in Figure S3). These new signals were attributed to cyclobutyl derivatives, suggesting an intermolecular [2 + 2] cycloaddition, forming pyrenyl‐ and pyridinyl‐substituted cyclobutanes. The formation of substituted cyclobutanes is confirmed by the UV spectra, on which the characteristic absorption bands of pyrene can be observed between 300 and 380 nm (Figure 4a), associated with the loss of the conjugation with the pyridine through the vinyl bond. Moreover, the formation of cyclobutanes following the irradiation of PyPe at 450 nm is documented in the literature,^[^ ^27^ ^]^ displaying the same 1H NMR shifts as those obtained in this work with irradiation at 400 nm. They were then identified as syn‐head‐to‐tail (5.2 and 6.0 ppm) and syn‐head‐to‐head (4.5 and 5.1 ppm) products (Figure 4c).

**Figure 5:**
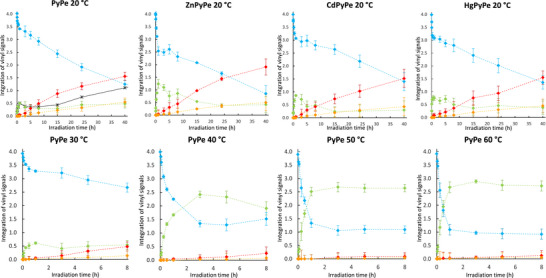
^1^H NMR monitoring of the irradiation of **PyPe**, **ZnPyPe**, **CdPyPe**, and **HgPyPe** at different temperatures. Plot of the integration of signals at 7.6 + 8.7 ppm (vinyl E, blue curve), 7.0 + 7.7 ppm (vinyl Z, green curve), 5.2 + 6.0 ppm (cyclobutyl syn htt, red curve), and 4.5 + 5.1 ppm (cyclobutyl syn hth, orange curve), based on a total integral from 4.0 to 11.0 ppm being fixed to 30 protons, representing 2 **PyPe** or 1 dimer. The black curve in first graph represents Z / (Z + E) ratio. Error bars represent the difference to 4 protons.

A plot of the characteristic proton NMR signals of each of the compounds (Figure 5, top left) shows that the ratio Z/(*Z *+ *E*) overlaps at the beginning of the experiment with the percentage of Z and that both curves split after 5 to 8 hours, indicating that the proportion of dimers starts to be significant at that time. Moreover, the predominance of the *syn htt* dimer is also observed, whose proportion continuously increases up to 39 % after 40 hours of irradiation (together with 31 % of *E‐*, 12 % of *Z‐*, and 14 % of *syn hth*). Indeed, in the *syn hth*, the two pyrene groups are both in front of each other, causing likely some steric hindrance. The intermediate of the dimerization is therefore strongly destabilized, and the reaction could then be disfavored.

### 2.3 Kinetics

The same experiments at different temperatures show a strong evolution of the final percentage of each compound (Table 2, and Figure 5). First, the increase in temperature leads to an increase in *E*‐*Z* isomerization kinetics as it allows a faster bond rotation along the vinyl linker. From the computation of kinetics constants and the application of the Arrhenius law, the activation energy was calculated to be 3.08 kJ.mol^−1^. This value is in the same range as different publications on similar compounds.^[^ ^22^, ^28^, ^29^ ^]^ As a result, the proportion of *Z* isomer versus *E* increases (after 8 hours, the Z/E ratio is 0.12 at 20 °C, and 2.96 at 60 °C). Effects on absorption and emission properties can be seen in Figure S4.

**Table 2:** Percentage of all molecules present in irradiated PyPe solutions, from ^1^H NMR measurements.

		20 °C	30 °C	40 °C	50 °C	60 °C
*E*	After 1 hour	86	82	56	33	27
	After 8 hours	73	67	38	27	23
*Z*	After 1 hour	13	15	42	63	67
	After 8 hours	7.5	14	48	66	68
*syn htt*	After 1 hour	1.3	1.3	1.0	0.0	0.8
	After 8 hours	12	12	6.3	2.3	3.0
*syn hth*	After 1 hour	0.0	0.5	0.0	0.0	0.3
	After 8 hours	3.2	4.0	2.0	1.0	1.3
*k*_EZ_	10^−7^ M.s^−1^	3.84	3.97	4.75	5.65	6.39
*k*_dim_	10^−9^ M.s^−1^	4.94	5.52	2.86	1.22	1.49

In parallel, a net decrease in dimer formation is observed with temperature. Indeed, at higher temperature constraint cycles, such as cyclobutyl, are more difficult to form, and whereas proportions are similar after 1 hour (1.3 vs. 0.8% for *syn htt*), after 8 hours of irradiation, up to 15% of *syn htt* is formed at 20 °C but only 3.1% at 60 °C. At 40 °C, which is around the usual temperature for bioimaging experiments, the proportion of dimers is divided by 2 compared to 20 °C (7% after 8 hours).

### 2.4 Complexes

Coordination compounds between *E‐* **PyPe** and metal iodide salts ZnI_2_, CdI_2_, and HgI_2_ were synthesized, resulting, respectively, in the coordination compounds **ZnPyPe**, **CdPyPe**, and **HgPyPe**.^[^ ^21^ ^]^ The metal ion is in each case coordinated by two **PyPe** ligands. Its coordination sphere is completed by two iodide anions, resulting in a distorted tetrahedral geometry.^[^ ^21^ ^]^

**Figure 6:**
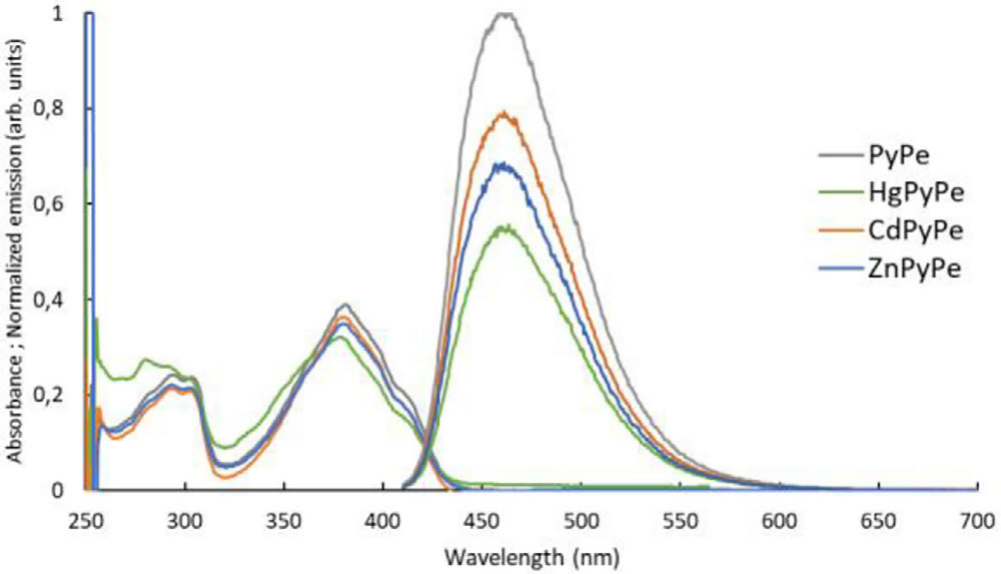
Absorbance and emission spectra of **PyPe** (22 µM) and its complexes (11 µM of complexes, resulting in 22 µM of ligand).

Absorption and emission studies of these complexes were performed. Comparison of the results showed that absorption and emission maxima were identical, but intensities varied. Indeed, complexes have slightly lower molar absorption coefficients (**PyPe**: 35 162 L*mol^−1^*cm^−1^, up to 28 693 L*mol^−1^*cm^−1^ for **HgPyPe**), resulting in more different emission intensities (from 1.00 for **PyPe** to 0.55 for **HgPyPe**) (Figure 6 and Table 3).

**Table 3:** Absorption and emission properties of **PyPe** and its complexes.

	PyPe	ZnPyPe	CdPyPe	HgPyPe
Absorption maxima (nm)	380	381	380	379
Molar absorption coefficient (L*mol^−1^*cm^−1^) at abs. max.	17 580 (35 162)^[^ ^a^ ^]^	31 639	33 005	28 693
Emission maxima (nm)	459	461	461	460
Normalized emission intensity (arb. units)	1.00	0.69	0.79	0.55

^[a]^ in brackets, multiplied by 2 to compare with constant ligand concentration

Whereas complexation reduces the degree of freedom of **PyPe**, the study of the photochemical capacities of **ZnPyPe, CdPyPe**, and **HgPyPe** showed that the complexation and the nature of the d^10^ metal ion could influence the isomerization and dimerization rates. Hence, irradiation monitoring of complexes results after 40 h in a ratio *E:Z:synhtt:synhth* of, respectively, 23:11:52:14, 38:8:42:12, and 36:10:41:12 (33:12:41:14 for **PyPe** alone) (Figure 5).

First, it is observed that the dimerization appears more efficient for **ZnPyPe** than for **CdPyPe**, **HgPyPe,** and **PyPe** itself: After 40 hours of irradiation, 65.5% of **ZnPyPe** has dimerized (51.9% as *syn hth*, 13.6% as *syn htt*) against 53.4, 53.4, and 59.0%, respectively, for the other compounds. Second, the kinetics of **CdPyPe** and **HgPyPe** (3.8 and 3.5*10^−9^ M*s^−1^ dimers are produced) are slower than for **ZnPyPe** and **PyPe** itself (5.2 and 4.9*10^−9^ M*s^−1^). At first glance, these observations could be surprising, as the molar absorption coefficient of **CdPyPe,** for instance, is closer to **PyPe** than that of **ZnPyPe** (94% vs. 90%), whereas **CdPyPe** and **HgPyPe** are more different (87%).

However, in isomerization and dimerization, the position of the ligands seems to have more importance than (slight) variations of molar absorptivity. The precise geometry of both ligands within the complex could be involved in this behavior and explain the observed differences. Indeed, the formation of the dimers requires that two **PyPe** molecules adopt the correct configuration for a [2 + 2] cycloaddition. The metal ion‐pyridine bond increases along the *d* ^10^ group with 2.08 Å for the zinc complex, 2.29 Å for the cadmium complex, and finally 2.42 Å for the mercury complex. Hence, two reasons could explain the different behavior of the complexes: First, the zinc‐ion–pyridine length corresponds exactly to the ideal configuration for a [2 + 2] addition, (slightly) favoring the formation of dimers from **ZnPyPe**. Second, the evolution of the metal‐ion–pyridine length is itself linked to the charge density of metal ions, depending on their ionic radius, which increases from zinc to mercury (ionic radius of 0.74, 0.95, and 1.02 Å, respectively).^[^ ^30^ ^]^ A bond valence sum calculation helped to estimate the bond efficiency (Table 4) around each metal ion. It turns out that the bond valence for the M‒I bond becomes stronger from zinc to mercury, while the relative contribution to charge compensation of the **PyPe** decreases in that order. The Hg‒N bond is thus the weakest of the three complexes, meaning that **HgPyPe** is the complex where **PyPe** possesses the greatest degree of freedom, so with the closest behavior of **PyPe** alone. **CdPyPe** is then in an intermediate case, whereas **ZnPyPe** is the complex where **PyPe** has the smallest degree of freedom and displays a different behavior than **PyPe**.

**Table 4:** Sum‐up of the quantity of all molecules present in irradiated solutions, from ^1^H NMR measurements.

		PyPe	ZnPyPe	CdPyPe	HgPyPe
Ionic radius of the ion (pm) ^[^ ^30^ ^]^		‐	74	95	102
Distance between ion and **PyPe** nitrogen (Å)		‐	2.069(7) and 2.085(7)	2.30(1) and 2.29(1)	2.418(4) and 2.424(4)
Contribution of N and I to M^2+^ coordination		‐	0.40 and 0.61	0.38 and 0.73	0.34 and 0.87
Bond Valence Sum			1.99	2.27	2.42
Percentage of E	After 1 hour	86	64	77	77
	After 5 hours	79	65	75	72
	After 40 hours	31	21	35	34
Percentage of Z	After 1 hour	13	31	22	19
	After 5 hours	7.8	19	11	18
	After 40 hours	12	11	8	10
Percentage of Z/(Z + E)	After 1 hour	13	33	22	20
	After 5 hours	8.9	23	13	20
	After 40 hours	28	33	18	22
Percentage of *syn htt*‐dimer	> 2 %	3h	3h	3h	1h
	After 40 hours	39%	48%	38%	39%
Percentage of *syn hth‐*dimer	> 2 %	8h	8h	8h	8h
	After 40 hours	14%	13%	11%	11%
Deviation from 4 protons (%)	After 1 hours	0.8	4.2	0.8	2.0
	After 5 hours	3.8	4.8	5.3	6.2
	After 40 hours	4.0	8.0	8.8	6.5

### 2.5 Bioimaging

**Figure 7:**
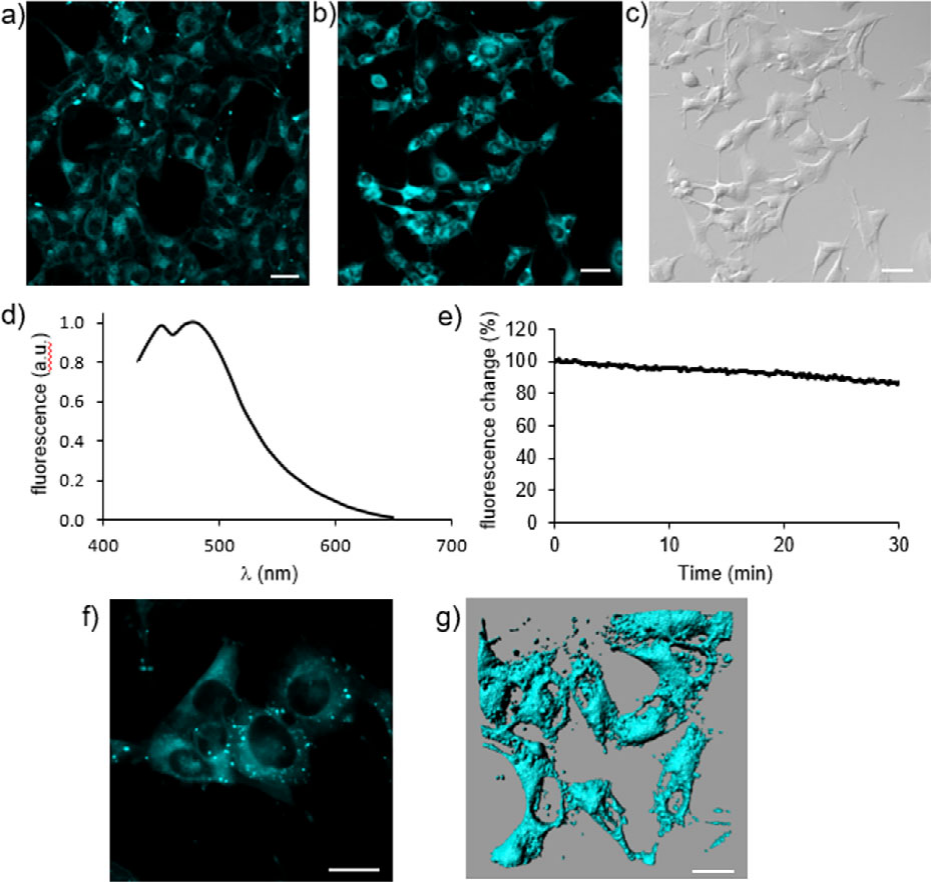
a) Low magnification fluorescence image of **PyPe** after loading in HEK cells b) **PyPe** loaded in the B16‐OVA melanoma cell line; c) Corresponding DIC image; d) **PyPe** fluorescence emission spectrum excited at 405 nm, showing peaks at 465 nm (**PyPe**) in situ; e) Time course of intracellular fluorescence acquired at 0.1 Hz demonstrating the high level of cellular retention of the dyes and the low rate of photobleaching f) High‐magnification fluorescence image of B16‐OVA cells loaded with **PyPe** showing cytosolic staining; g) 3D reconstruction of **PyPe** fluorescence in B16‐OVA. Scale bars: a‐c: 50 um; f, g: 20 um.

The blue emission of **PyPe**, associated with its photostability and its scalable synthesis protocol, makes this dye a promising candidate as a dye for bioimaging. Moreover, the absence of functional groups makes it particularly robust in buffer systems. It was readily taken up by HEK and B16‐OVA cells, using standard procedures within 1 hour at concentrations of 10 µM, and led to homogeneous dye intake by the cell population (Figure 7a–c). The peak of in situ fluorescence emission spectra, when excited at 405 nm, was at 465 nm (Figure 7d). No significant excitation occurred at other wavelengths commonly used in fluorescence microscopy (458, 488, 514, 561 nm), making the dye suitable for multispectral imaging. While scanning at 0.1 Hz over 30 minutes, using a laser power of 30 µW at the objective, the signal intensity remained higher than 90% compared to t = 0 (Figure 7e), indicating the long‐term stability of the dye in living cells and the low rate of photobleaching. At higher magnification, loading appeared cytosolic with some partitioning in subcellular compartments, sparing the nucleus (Figure 7f, g). No morphological signs of cell toxicity were observed during the duration of experiments at the used concentrations (up to 50 µM).

**Figure 8:**
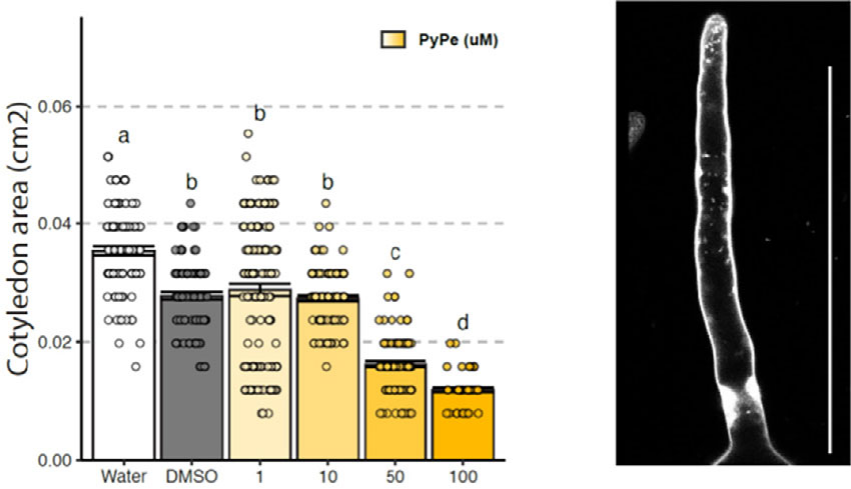
Left: Cotyledon area of Arabidopsis seeds growing on media supplemented with *E‐*PyPe after 8 days, concentration in µM. Right: Confocal microscopy of Arabidospis root tips after PyPe staining. Excitation at 405 nm and emission recorded from 425 to 525 nm. Scale bar represents 100 µm.

Complementary assays were realized in plant tissues on Arabidopsis seedlings. Cytotoxicity assays were performed, and **PyPe** was tested for staining of root tissues (Figure 8 and Figure S5). To evaluate the toxicity of this compound, Arabidopsis seedlings growing on media containing **PyPe**, were analysed for two parameters: primary root growth and cotyledon development (Figure 8 and Figure S6). We found that Arabidopsis tolerates a concentration of **PyPe** up to 10 µM without affecting growth. At the concentration of 100 µL, seedlings showed reduced cotyledon size and shorter roots (Figure 8). Arabidopsis root tissues were well stained by **PyPe** in the elongation and maturation zone, with a clear observation of cell contour and partially diffusing into the cytoplasm. We observed that the staining was less efficient in the root meristem region, where the stem cell niche and highly dividing root cells are found (Figure S5).

As toxicity starts to be observable at 10 µM on Arabidopsis root lengths, we performed staining with only 1 µM of **PyPe**. At this concentration, however, photobleaching is faster than at the 10 µM working concentration used for scanning HEK‐cells. While scanning at 0.2 Hz over 30 minutes, using a laser power of about 6 mW at the objective (i.e., 40 times stronger irradiation than HEK‐cells), about 60% of fluorescence emission is lost (Figure S7).

To test which compartment in the root cells is stained, we performed two sets of experiments. First, we used fresh protoplasts from the leaves of Nicotiana benthamiana and stained them with 1µM of the dye. The protoplasts lack a cell wall. We could observe that **PyPe** was not staining the plasma membrane, but it was partially diffusing into the cytosol. Second, we used Arabidopsis roots for double staining with **PyPe** and FM4‐64, which is a known plasma membrane dye that undergoes endocytosis (Figure S8). Two dyes labelled the cell counter. To distinguish if **PyPe** stains the plasma membrane or cell wall, the plasmolysis with NaCl solution (0.5 M) was performed. Following plasmolysis, the plasma membrane labelled with FM4‐64 disconnected from the cell wall, labelled with **PyPe**. These two sets of experiments demonstrate that in the plant cells, **PyPe** stains the cell wall and diffuses into the cytoplasm but does not label the plasma membrane.

## 3 Conclusion

In summary, the **PyPe** is a dye compound presenting exceptional photochemical properties. The variation of some parameters, such as temperature and complexation, enables to get different photoreactivity, either the dimerization or the switch between *E* and *Z*‐configurations, as exemplified in solid‐state structures of their coordination compounds and during irradiation experiments in solution.

**PyPe** fits well for bioimaging applications, both in animal and plant cells, with a strong stability over a long time scale of light irradiation and almost no photobleaching occurring during bioimaging experiments at 10 µM. During 30 minutes exposition to confocal microscopy laser light (0.1 Hz, 30 µW at the objective), the dye molecule provided excellent stability emitting blue light, and no emission at other common wavelengths. Furthermore, the fluorophore was easily taken up by cells, being nontoxic at concentrations up to 10 µM. Since, in particular blue‐emitting, photo‐stable dyes are very rare, it contributes to the pool of highly stable dyes that can be used in parallel, avoiding interference with other dye compounds. A future potential application might therefore be cell sorting. Coupled to targeting moieties like biotin, Streptavidin, or Avidin, it might also be a valuable dye for specific histological staining experiments.

## Supporting Information

The authors have cited additional references within the Supporting Information.^[^ ^31^, ^36^ ^]^

## Conflict of Interest

The authors declare no conlict of interest.

## Supporting information



Supporting Information

Supporting Information
