# Supplementary material for: Coping with the Blues: Simple and Photo‐Stable Dye for Long‐Term Live‐Cell Imaging
Source: Chemistry. 2025 Oct 17;31(70):e01360. doi: 10.1002/chem.202501360 (PMC12712751; doi:10.1002/chem.202501360)
Supplement: Supplementary file 1 — Supporting Information [file CHEM-31-e01360-s002.docx]

**Supporting Informations**

Procedures

**General**

1-pyrenecarboxaldehyde and 4-picoline were obtained from Fluorochem. Phosphoryl chloride was purchased from Aldrich (Saint-Louis, MO, US), di-isopropylamine from Acros Organics (Fisher Scientific Gmbh, Reinach, Switzerland), pyridine from Fisher Scientific Gmbh (Reinach, Switzerland), and all other chemicals were purchased from Sigma-Aldrich (Saint-Louis, MO, US) and used as received if not otherwise stated.

NMR spectra were recorded on a Avance III 400 MHz spectrometer (Bruker, Billerica, MA, US) at room temperature with CDCl_3_ or *d*_6_-DMSO as solvents. Mass spectra were recorded on a Ion-Trap ESI-MS (Bruker, Billerica, MA, US). Fluorescence spectra were measured on a Perkin Elmer instrument LS50B (Wellesley, MA, US), and absorption spectra were collected on a Perkin Elmer UV/VIS Lambda 25 spectrometer (Wellesley, MA, US).

1-(Pyren-1-yl)-2-(Pyridin-4-yl)Ethan-1-ol (1) Synthesis


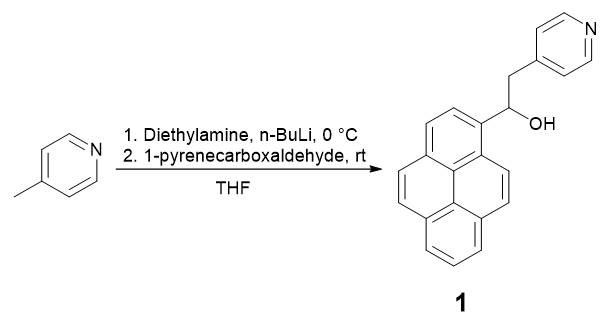


10 mL of ice-cold dry THF was placed in a flask under argon, and 4-picoline (490 µL, 5 mmol, 1.0 eq) was added. Diisopropylamine (665 µL, 5 mmol, 1.0 eq), *n*-BuLi in hexane (3.3 mL, 1.6 M, 5 mmol, 1.0 eq), and dry THF (2 mL) were mixed into a dropping funnel, and the mixture was added dropwise at 0 °C over 25 min. The resulting mixture turned from yellowish to red. At the end of addition, the dropping funnel was rinsed with 2 mL of dry THF, and the dark red solution was stirred for 1 h at 0–5 °C.

1-pyrenecarboxaldehyde (1.1 g, 5 mmol, 1.0 eq) was solubilized in 7 mL of dry THF under argon, and this green-brown solution was added dropwise to the previous one at 20 °C. Immediately after addition, a yellowish precipitate formed, and the solution turned brown. The resulting mixture was then stirred at room temperature overnight.

The orange solution was quenched with crushed ice, and after 10 min, THF was evaporated under reduced pressure. Then, 15 mL of distilled water was added to the yellow mixture, and the aqueous phase was extracted three times with about 20 mL of DCM. The combined organic layers were evaporated under reduced pressure, and a yellow oily solid was obtained, characterized by ^1^H NMR (400 MHz, Chloroform-d) δ 8.50–8.45 (m, 2H), 8.31 (d, J = 9.3 Hz, 1H), 8.23–8.01 (m, 9H), 7.21–7.13 (m, 2H), and 6.03 (dd, J = 7.8, 5.0 Hz, 1H), 3.37–3.23 (m, 2H). ^1^H NMR was not interpretable, but ESI-MS corresponded to the targeted product (calc. 323.4 *m*/*z*, found M+H^+^ 324.1 *m*/*z*), so the crude was used without further purifications.

(E)-4-(2-(Pyren-1-yl)Vinyl)Pyridine (PyPe) Synthesis


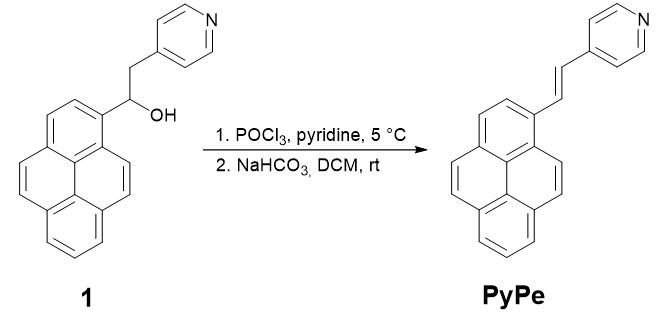


Compound **1** was sonicated in 10 mL of pyridine under argon to obtain a stable suspension. Then, phosphoryl chloride (700 µL, 7.5 mmol, 1.5 eq) in 6 mL pyridine was added dropwise at 5 °C. After addition, the resulting red suspension was stirred at room temperature for 3 h. Then, the mixture was quenched with some pieces of crushed ice (vapors were formed) and stirred for 10 min. The solvent was evaporated under reduced pressure, forming a red sludge.

About 30 mL each of DCM and acidified water (HCl, pH < 1) were added, and the organic layer was washed with acidified water. The non-dissolved solid was kept with the organic layer. The organic layer in DCM and red solid were vigorously shaken with 20 mL of sodium bicarbonate in water (1M), resulting in the formation of a beige precipitate. When all red color disappeared, the two layers were separated, and the aqueous layer was washed with DCM. The solvent was then evaporated under reduced pressure. From this, 1.0 g of beige solid (3.4 mmol, 68% total yield) was obtained, characterized by ^1^H NMR (400 MHz, DMSO-d_6_) δ 8.82 (d, J = 9.4 Hz, 1H, pyrene), 8.69 (d, J = 16.2 Hz, 1H, vinyl), 8.65–8.61 (m, 2H, pyridine), 8.56 (d, J = 8.2 Hz, 1H, pyrene), 8.36–8.32 (m, 3H, pyrene), 8.30 (d, J = 9.4 Hz, 1H, pyrene), 8.22 (m, 2H, pyrene), 8.10 (t, J = 7.6 Hz, 1H, pyrene), 7.87–7.82 (m, 2H, pyridine), 7.56 (d, J = 16.1 Hz, 1H, vinyl), and ESI-MS (calc. 305.1 *m*/*z*, found M+H^+^ 306.2 *m*/*z*).

**Crystallization**

[Zn(*E-*PyPe)_2_Cl_2_]∙2DCM **1**: A solution of PyPe at 10 mM in DCM (30.5 mg, 100 µmol ) was prepared and mixed with 10 mL of a solution of ZnCl_2_ at 5 mM in methanol (6.8 mg, 50 µmol). After 1 h, an orange precipitate was formed, which was collected, washed with n-pentane, and dried. Crystals of the compound **1** were obtained by self-diffusion of component over one week. Yield is 64%. EA: (calculated) C, 67.85; H, 3.88; Cl, 17.04; N, 3.37; Zn, 7.86%; (found) C, 67.10; H, 3.67; N, 3.20%.

A suitable crystal was selected and mounted on a loop with oil on a Stoe IPDS 2 diffractometer. The crystal was kept at 250(2) K during data collection. Using Olex2 [31], the structure was solved with the SHELXT [32] structure solution program using Intrinsic Phasing and refined with the SHELXL [33] refinement package using Least Squares minimisation. Crystal Data for C_94_H_64_Cl_8_N_4_Zn_2_ (*M*=1663.83 g/mol): monoclinic, space group C2/c (no. 15), *a* = 15.0774(13) Å, *b* = 11.0294(8) Å, *c* = 23.639(2) Å, *β* = 97.002(7)°, *V*= 3901.7(6) Å^3^, *Z* = 2, *T* = 250(2) K, μ(Mo Kα) = 0.940 mm^-1^, *Dcalc* = 1.416 g/cm^3^, 24463 reflections measured (3.472° ≤ 2Θ ≤ 50.372°), 3472 unique (*R*_int_ = 0.1071, R_sigma_ = 0.0477) which were used in all calculations. The final *R*_1_ was 0.0684 (I > 2σ(I)) and *wR*_2_ was 0.1658. CCDC-2031641.

[Zn(*E-*PyPe)(Z-PyPe)Cl_2_]∙2DCM **2**: Compound **2** was synthesized by exposure of a solution of **1** (1 mg ca) in MeOH-DCM (1:1) with 400 nm light (Hg short arc lamp 300 Watt light source, using band-pass filter 400 nm, 25 nm FWHM) for 0.5 h. Complexes were obtained as small plate-needles barely suitable for single crystal diffraction. EA was not performed due to very small quantitate yield of complexes.

A crystal was selected and mounted on a loop with oil on a Stoe IPDS 2 diffractometer. The crystal was kept at 250(2) K during data collection. Using Olex2 [31], the structure was solved with the SHELXT [32] structure solution program using Intrinsic Phasing and refined with the SHELXL [33] refinement package using Least Squares minimisation. Crystal Data for C_46_H_30_Cl_2_N_2_Zn (*M*=746.99 g/mol): monoclinic, space group P2_1_/c (no. 14), *a* = 21.405(4) Å, *b* = 9.7675(13) Å, *c* = 16.846(3) Å, *β* = 97.357(14)°, *V*= 3493.1(10) Å^3^, *Z* = 4, *T* = 250(2) K, μ(MoKα) = 0.894 mm^-1^, *Dcalc* = 1.420 g/cm^3^, 20283 reflections measured (3.838° ≤ 2Θ ≤ 50.648°), 6070 unique (*R*_int_ = 0.2131, R_sigma_ = 0.2105) which were used in all calculations. The final *R*_1_ was 0.0684 (I > 2σ(I)) and *wR*_2_ was 0.2164. CCDC-2433742.

[ZnPyPe_2_I_2_] **ZnPyPe**: A solution of PyPe at 10 mM in dry THF (30.5 mg, 100 µmol) was prepared, poured into a vial, and covered with 10 mL of a solution of zinc iodide at 5 mM in dry THF (16.0 mg, 50 µmol). The vials were sealed and left in the dark for three weeks. The supernatant was then removed, and the obtained orange needles (25.1 mg, 27 mmol, 54%) were washed with a small quantity of chloroform and analyzed via X-Ray diffraction. EA: (calculated) C, 59.41; H, 3.01, N, 3.25%; (found) C, 57.80; H, 2.93; N, 2.72.

A suitable crystal was selected and mounted on a loop with inert oil on a STADIVARI diffractometer. The crystal was kept at 250(2) K during data collection. Using Olex2 [31], the structure was solved with the SHELXT [32] structure solution program using Intrinsic Phasing and refined with the SHELXL [33] refinement package using Least Squares minimization. Crystal Data for C_46_H_30_I_2_N_2_Zn (*M* =929.89 g/mol): monoclinic, space group *P*2_1_/n (no. 14), *a* = 7.1432(2) Å, *b* = 14.9839(3) Å, *c* = 33.7701(8) Å, *β* = 91.979(2)°, *V* = 3612.36(15) Å^3^, *Z* = 4, *T* = 250 K, μ(Cu Kα) = 14.638 mm^−1^, *Dcalc* = 1.710 g/cm^3^, 6410 reflections measured (5.24° ≤ 2Θ ≤ 136.31°), 6410 unique (*R*_int_ = /, R_sigma_ = 0.0647, twin 0.9694(14) : 0.0307(14)), which were used in all calculations. The final *R*_1_ was 0.0690 (I > 2σ(I)) and *wR*_2_ was 0.2103. CCDC*-*2290405*.*

[CdPyPe_2_I_2_] **CdPyPe**: A solution of PyPe at 8 mM in chloroform/acetonitrile 1:5 (19.5 mg, 64 µmol) was prepared. Following this, 8 mL was poured into a vial and layered with 2.4 mL of a solution of cadmium iodide at 4 mM in methanol (23.4 mg, 32 µmol). The vials were sealed and left in the dark for three weeks. The supernatant was then removed, and the obtained orange needles (21.5 mg, 22.1 µmol, 69%) were washed with a small quantity of ethanol and analyzed via X-Ray diffraction. EA: (calculated) C, 56.55; H, 3.10, N, 2.87% ; (found) C, 56.27; H, 2.73; N, 2.70.

A suitable crystal was selected and mounted on a loop with oil on a Stoe IPDS II diffractometer. The crystal was kept at 250(2) K during data collection. Using Olex2 [31], the structure was solved with the SHELXT [32] structure solution program using Intrinsic Phasing and refined with the SHELXL [33] refinement package using Least Squares minimization. Crystal Data for C_46_H_30_CdI_2_N_2_ (*M* =976.92 g/mol): monoclinic, space group *P*2_1_/n (no. 14), *a* = 7.1842(3) Å, *b* = 15.2855(5) Å, *c* = 33.4198(17) Å, *β* = 91.990(4)°, *V* = 3667.8(3) Å^3^, *Z* = 4, *T* = 250(2) K, μ(MoKα) = 2.315 mm^−1^, *Dcalc* = 1.769 g/cm^3^, 47945 reflections measured (2.44° ≤ 2Θ ≤ 53.7°), 7825 unique (*R*_int_ = 0.0740, R_sigma_ = 0.0390), which were used in all calculations. The final *R*_1_ was 0.0391 (I > 2σ(I)), and *wR*_2_ was 0.1089. CCDC-2290406.

[HgPyPe_2_I_2_] **HgPyPe**: A solution of PyPe at 20 mM in chloroform (61.1 mg, 192 µmol) was prepared and 10 mL was poured into a vial and layered with 2.4 mL of a solution of mercury (II) iodide at 40 mM in dry THF (43.6 mg, 96 µmol). The vials were sealed and left in the dark for three weeks. The supernatant was then removed, and the obtained thin red needles (46.0 mg, 44 µmol, 45%) were washed with a small quantity of ethanol and analyzed via X-Ray diffraction. EA: (calculated) C, 51.87; H, 2.84, N, 2.63% ; (found) C, 51.90; H, 2.47; N, 2.42.

A suitable crystal was selected and mounted on a loop with oil on a Stoe IPDS II diffractometer. The crystal was kept at 250(2) K during data collection. Using Olex2 [31], the structure was solved with the SHELXT [32] structure solution program using Intrinsic Phasing and refined with the SHELXL [33] refinement package using Least Squares minimization. Crystal Data for C_46_H_30_HgI_2_N_2_ (*M* =1065.11 g/mol): monoclinic, space group *P*2_1_/n (no. 14), *a* = 7.1862(3) Å, *b* = 15.2968(4) Å, *c* = 33.2922(13) Å, *β* = 92.088(3)°, *V* = 3657.2(2) Å^3^, *Z* = 4, *T* = 250(2) K, μ(MoKα) = 5.933 mm^−1^, *Dcalc* = 1.934 g/cm^3^, 40578 reflections measured (2.45° ≤ 2Θ ≤ 52.44°), 7249 unique (*R*_int_ = 0.0720, R_sigma_ = 0.0398), which were used in all calculations. The final *R*_1_ was 0.0298 (I > 2σ(I)), and wR_2_ was 0.0635. CCDC-2290407.

<https://www.ccdc.cam.ac.uk/structures/id=doi:10.1002/chem.202501360> 2290405-2290407 and 2433742 contain the supplementary crystallographic data for this paper. These data are provided free of chargeby the joint CambridgeCrystallographic Data Centre and Fachinformationentrum arlsruhe http://www.ccdc.cam.ac.uk/structures.

**Absorption and emission (Fig 6, Fig S1)**

Organic solvents: Solutions at 2.2 mM (PyPe) or 1.1 mM (coordination compounds) in solvent were prepared, and 20 µL were sampled and diluted in 2.0 mL of solvent. Solutions ere measured in 1 cm cells in UV-Vis spectroscopy (measured 250-800 nm, 996 nm per minute) and fluorimetry (λ_ex_ 405 nm, slit 2.5 nm, measured 410-800 nm, 100 nm per minute).

Aqueous solutions: 10 mg of PyPe were weighed in three vials, and stirred vigorously for 2h with 5 mL of respectively 1M NaOH solution, distilled water, and 1 M HCl solution. The mixtures were then let stand overnight, and 4 mL of supernatant were recovered for measures (measured as organic solvents). The residue was dried and weighted, but weight difference was into balance error.

**Irradiation monitoring**

A solution at 2.2 mM (PyPe) or 1.1 mM (coordination compounds) in d_6_-DMSO was prepared, and 600 µL were transferred in an NMR tube. The NMR tube was irradiated using a Xe-arc lamp 300-Watt source, and a band-pass filter centered at 405 nm, 25 nm FWHM. The NMR tube was measured in 1H NMR at regular intervals, and 20 µL were sampled. These 20 µL were diluted in 2.0 mL of DMSO, and measured in UV-Vis spectroscopy (measured 250-800 nm, 996 nm per minute) and fluorimetry (λ_ex_ 405 nm, slit 2.5 nm, measured 410-800 nm, 100 nm per minute).

NMR spectra were integrated from 4.0 to 11.0 ppm, and these integrals were attributed to 30 protons (corresponding to 2 PyPe or 1 dimer compound). The characteristic peaks of vinyl bond (E: 8.70 + 7.56 ppm ; Z: 7.69 + 7.03 ppm) and cyclobutyl (htt: 6.04 + 5.22 ppm ; hth: 5.12 + 4.51 ppm) were then integrated, and their relative intensity were compared.

**Testing compounds in cell cultures**

HEK-293 and B16-OVA cell lines were grown in Dulbecco’s modified Eagle’s medium containing 2 mM L-glutamine, 4500 mg/l D-glucose, and 110 mg/l sodium pyruvate, supplemented with 10% heat-inactivated fetal bovine serum (FBS) and maintained in 95% O2/5% CO_2_ at 37°C. They were plated 48h before experiments on uncoated glass coverslips and used at 50-70% confluence.

To load the dyes, cell cultures were incubated in a solution containing (mM): 135 NaCl , 5.4 KCl, 20 HEPES, 1.3 CaCl_2_ , 0.8 MgSO_4_, 0.78 NaH_2_PO_4_ , 20 glucose (pH 7.4), supplemented with 0.1% Pluronic F 127 (Thermo Fisher Scientific), in the presence of 10 μM of PyPe for 1h at 37 °C. For imaging, cultures were then transferred to a solution containing (mM): 160 NaCl, 5.4 KCl, 20 HEPES, 1.3 CaCl_2_ , 0.8 MgSO_4_ , 0.78 NaH_2_PO_4_ , 5 glucose (pH 7.4) and bubbled with air.

Fluorescence acquisitions were done on a Leica SP5 inverted laser scanning confocal microscope with either a 20x/0.7 or a 40x/1.3 Oil objective. Dye excitation was done using a 405 nm laser line. Fluorescence emission was collected either with a standard photomultiplier tube or with a spectral detector. To assess the loading of the probes in cells, single plane images or z-stacks at 1µm intervals were acquired. To monitor the fluorescent stability, acquisitions of image time-series were done in frame mode at a rate of 0.1 Hz with a 20x/0.7 objective (pixel size: 1x1 μm, pixel dwell time: 4.9 us/pixel, no averaging) with a laser power at the objective of 30 uW (AOTF set at 2%). Differential interference contrast (DIC) images of cell cultures where acquired using the light transmitted through the condenser.

Confocal images were processed with Fiji [34]. Fluorescence intensity over time traces were drawn from regions of interest and represented as relative fluorescence changes over the initial intensity. Fluorescence emission spectra were drawn from regions of interest and represented as relative fluorescence changes over the maximum intensity.Three-dimensional reconstructions of cells were performed from z-stacks using the Imaris software package (Oxford Instruments). Calculations and charts were done with Excel (Microsoft).

**Cytotoxicity on Arabidopsis**

Arabidopsis seeds were sterilized by submerging them in 70% ethanol for 5-10 minutes followed by quickly submerging in 100% ethanol before airdrying in sterile conditions until completely dry. Seeds were sown on square plates containing ½ MS (Duchefa), 0.8% agar (Duchefa), 1% sucrose (Roth), and adjusted to a pH of 5.6-5.7 with KOH, supplemented with increasing concentration of PyPe (1, 10, 50, and 100 µM) or DMSO as mock. After sowing, plates were kept in the dark at 4^o^C for at least 48 h before being transferred to a growth chamber at 22°C with 16h light/8h dark cycles for 8 days. Plates were placed vertically for root measurements and horizontally for cotyledon measurements. Plates were scanned with an Epson scanner at high resolution (600dpi) and root length and cotyledon size was measured using the simple-neurite-tracer tool in Fiji or the built-in elliptical selection tool, respectively [34, 35]. Statistical analyses were performed in R v4.2.2 within the Rstudio interface. Statistical differences between multiple groups of quantitative data were determined by ANOVA followed by a post hoc Tukey test using the agricolae package (cran.r-project.org/web/ packages/agricolae/index.html) with a confidence level alpha of 0.05.

**Staining of Arabidopsis seedlings**

Seedlings were fixed with 4% paraformaldehyde (Sigma) in 1x PBS for 1 h minimum. After washing with 1x PBS, samples were cleared in ClearSee solution [36]. Samples were stained with 10 µM PyPe for up to 1 h. Imaging was performed using a Leica TCS SP5 laser-scanning confocal microscope using a 405 nm laser line and detection at 425-525 nm.

To monitor the fluorescent stability, samples were stained with 1 μM PyPe as previously. Acquisitions of image time-series were done in frame mode at a rate of 0.2 Hz with a 40x/1.1 objective (pixel size: 0.3x0.3 μm, pixel dwell time: 1.41 μs/pixel, no averaging) with a laser power at the objective of 6 mW (AOTF set at 25%).


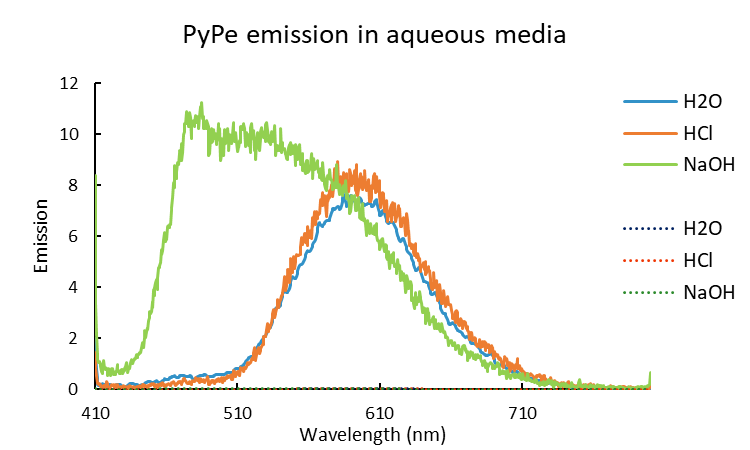

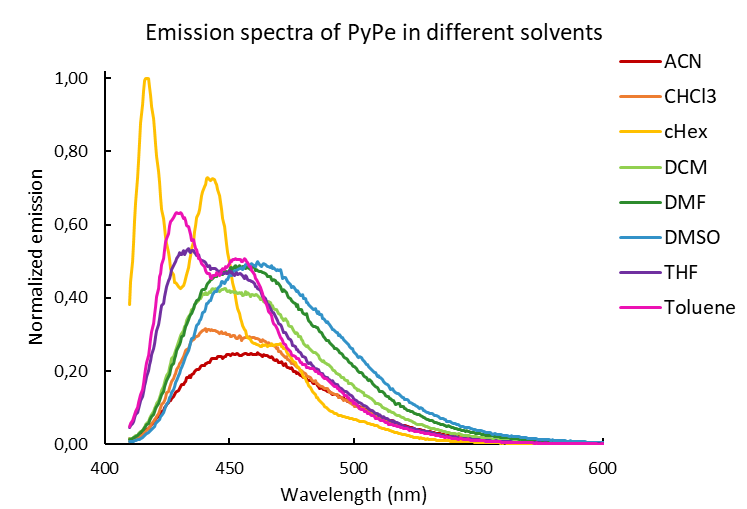

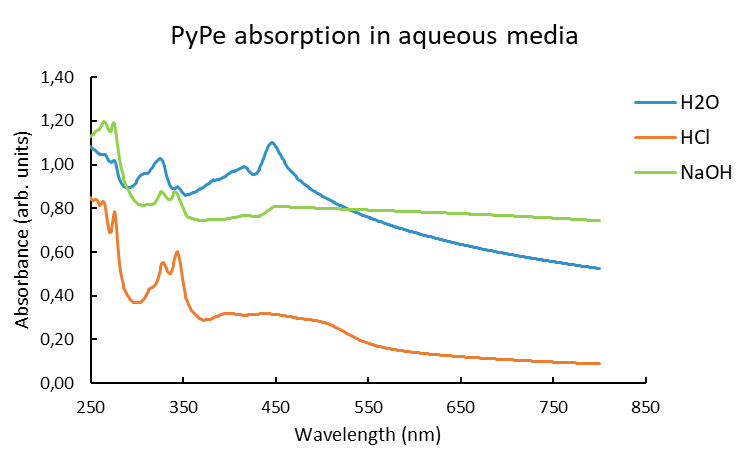

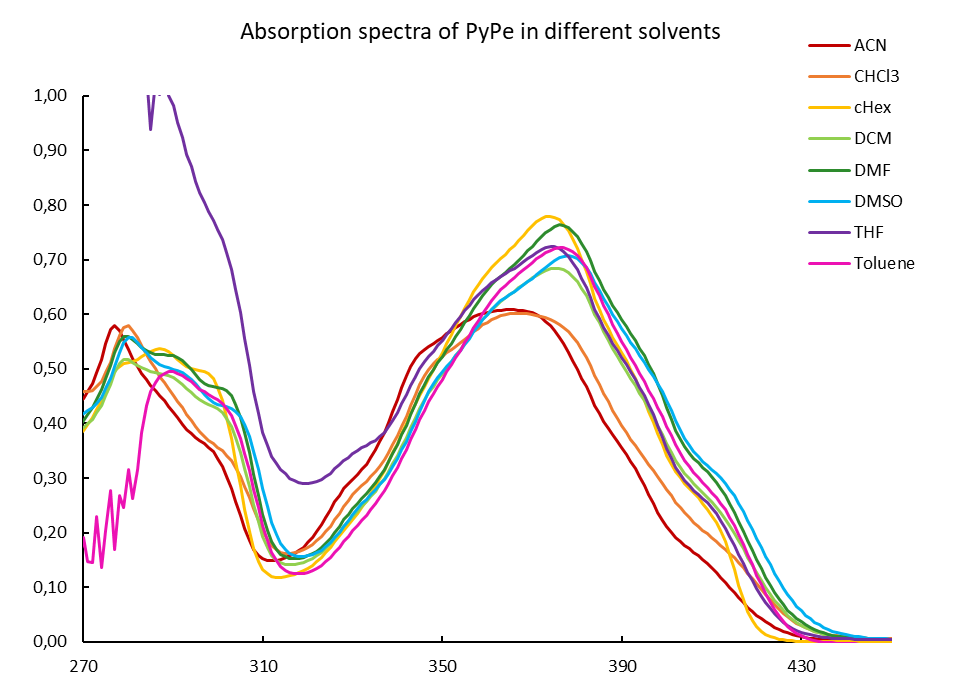
Fig S1: Absorption (top) and emission (bottom) of PyPe in different organic solvents at 25 μM (left) and in aqueous solutions (right). Concentration in aqueous solutions fall below the balance error. Emission in aqueous solutions are presented twice: normalized as in organic solutions for comparison (dotted lines, maxima around 0.01), and non-normalized (arb. units).

Table S1. Emission maxima and absorption, lifetime and quantum yield of PyPe.

| Solvent | λ^em^ _max_, nm^a^ | λ^abs^ _max_, nm | Molar absorption at 406 nm : ε^406^, L*mol^-1^*cm^-1^ | Lifetime,^b^ τ_avr._ns | Φ_f_, ^c^ | *k*_r_ [10^8^ s^-1^] | *K*_nr_ [10^8^ s^-1^] |
| --- | --- | --- | --- | --- | --- | --- | --- |
| DCM | 448 | 376, 280 | 11587 | 1.72 | 0.59 | 3.43 | 2.38 |
| THF | 433, 450 | 374 | 11120 | 2.26 | 0.51 | 2.26 | 2.17 |
| *c*-Hex | 416, 443 | 374, 287 | 10912 | 7.58 | 0.64 | 0.84 | 0.47 |
| DMF | 452 | 377, 280 | 13448 | 3.5 | 0.91 | 2.60 | 0.26 |
| Toluene | 452 | 376, 289 | 12402 | 2.25 | 0.88 | 3.91 | 0.53 |
| CH_3_CN | 456 | 365, 277 | 6557 | 2.07 | 0.92 | 4.44 | 0.38 |
| CHCl_3_ | 442 | 367, 280 | 8754 | 1.65 | 0.89 | 5.39 | 0.66 |
| DMSO | 459 | 378, 280 | 13917 | 1.03 | 0.75 | 7.32 | 2.40 |

^a^ λ_ex_ = 405 nm; ^b^ Multiexponential lifetime represented with τ_avr._,^c^ determined using integrating sphere.


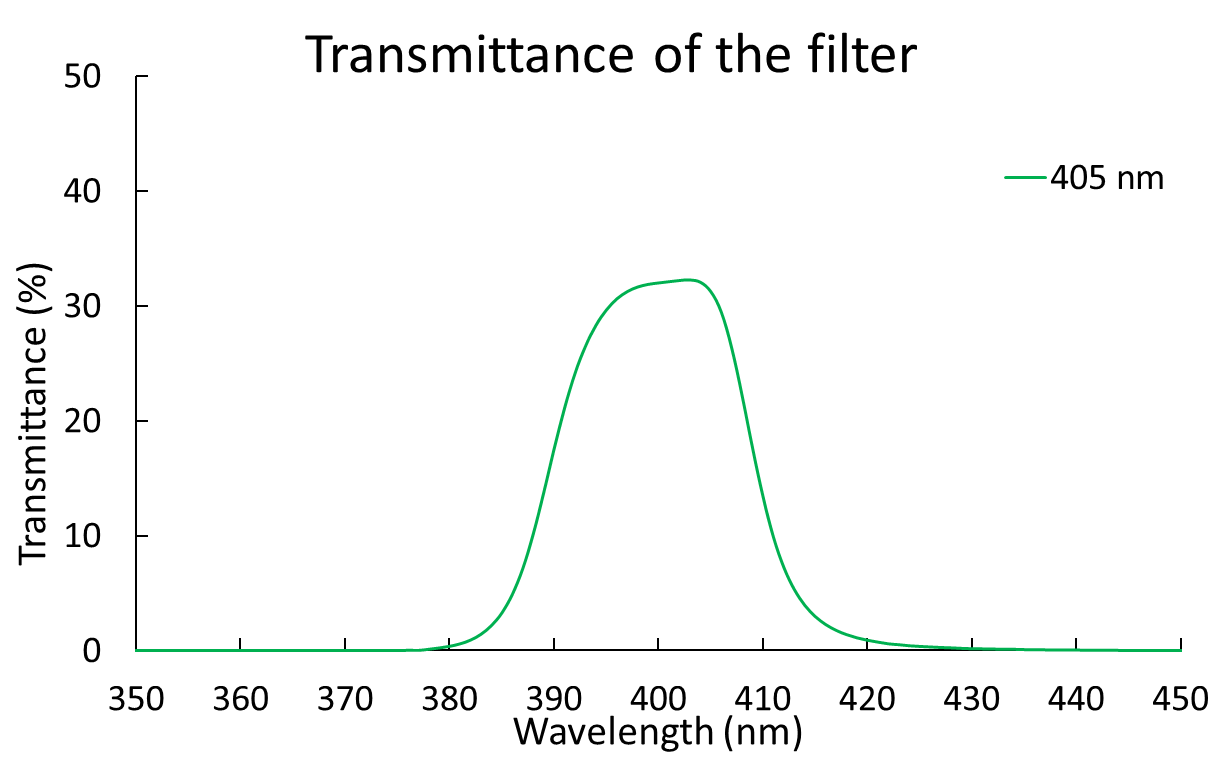

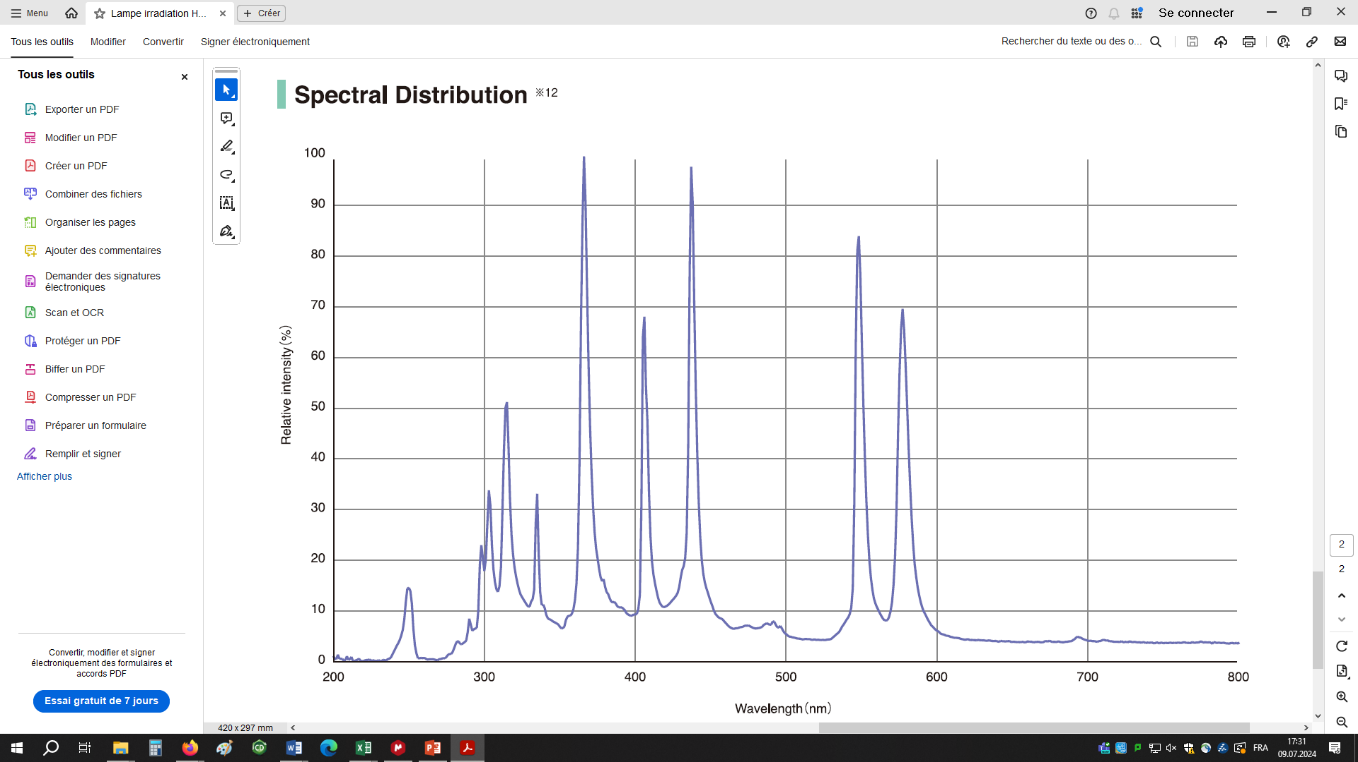
Fig S2: Left: Blank spectrum of the lamp, reference Ushio 5000506, USH-350DP Short Arc Lamp. Right: transmittance spectra of Andover Corporation Optical Filter AM-107605, 400FS20-25, SN39

8 h

5 h

3 h

1 h

30 min

15 min

5 min

3 min

0 min

• °• ° § §$ $

Fig S3: ^1^H NMR spectrum of PyPe after 8h of irradiation at 20 °C: *E-*PyPe ° , *Z-*PyPe • , *syn htt* § , *syn hth* $

Fig S4: Absorption and emission spectrum of *E-*PyPe during irradiation at 60 °C, resulting after 3h in a mixture of 75% Z-PyPe, 22% *E-*PyPe and 3% of dimer


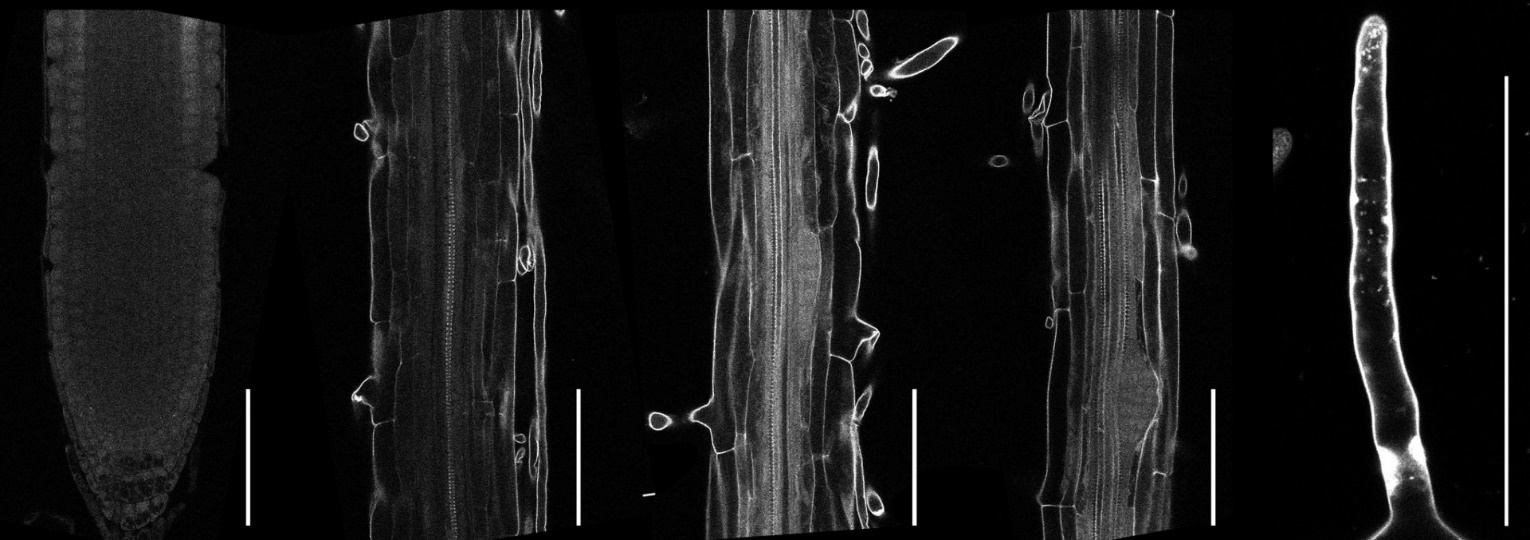


Fig S5: Confocal images of roots of Arabidopsis seedlings after fixation, clearing and staining with PyPe. From left to right: root tip, early elongation, late elongation, mature root, root hair. Scale bar represents 100 µm. Excitation 405 nm, emission 425-525 nm.

 a)

b) c) d) e)


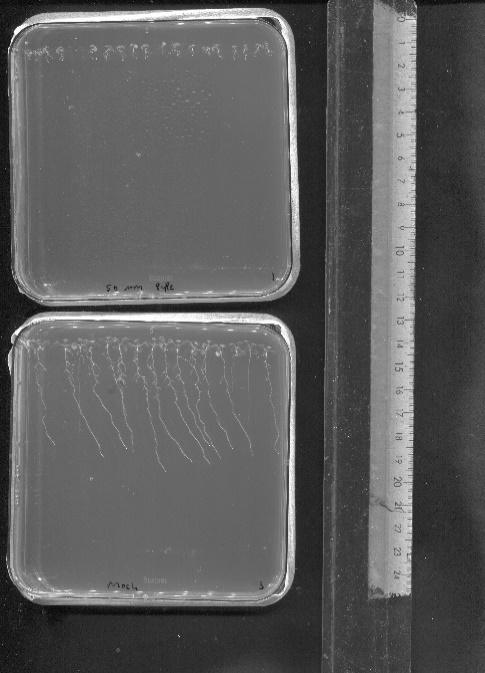

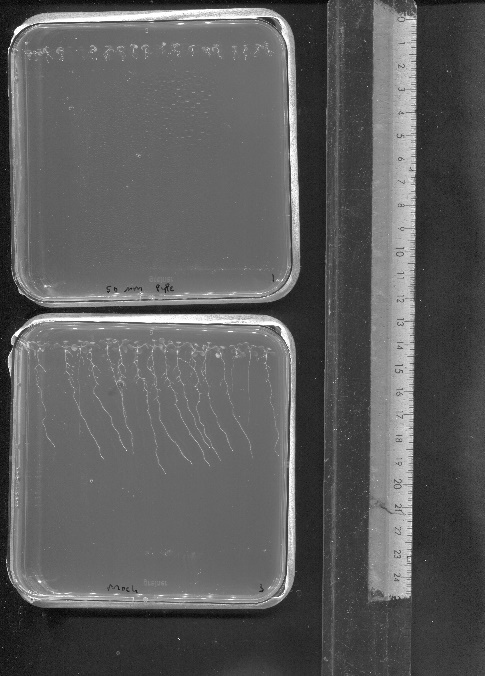

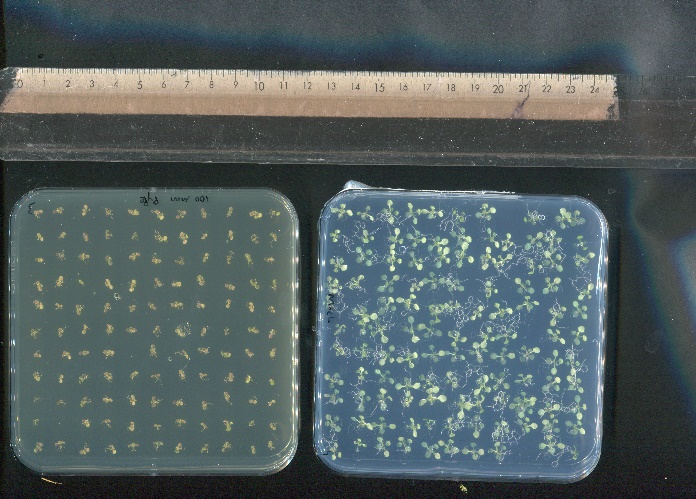

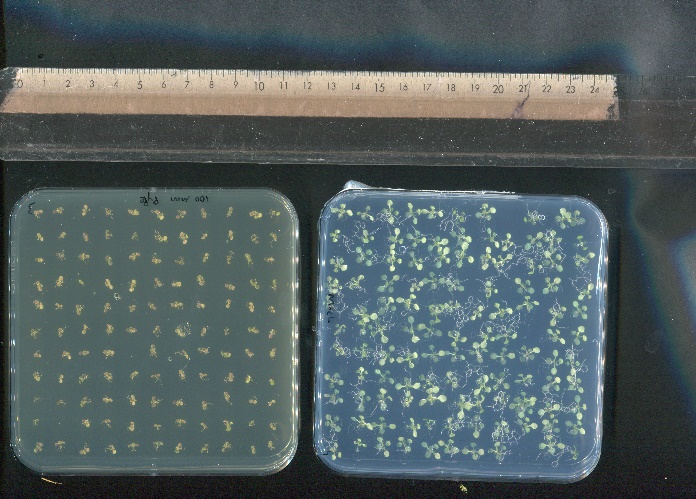


Fig S6: Toxicity assays of PyPe on Arabidopsis seeds. a) Measure of root length (left) and leaves area (right) in presence of increasing concentration of PyPe. b) Details of root length without PyPe and c) with 50 µM of PyPe. d) Details of cotyledon area without PyPe and in presence of 100 µM of PyPe.


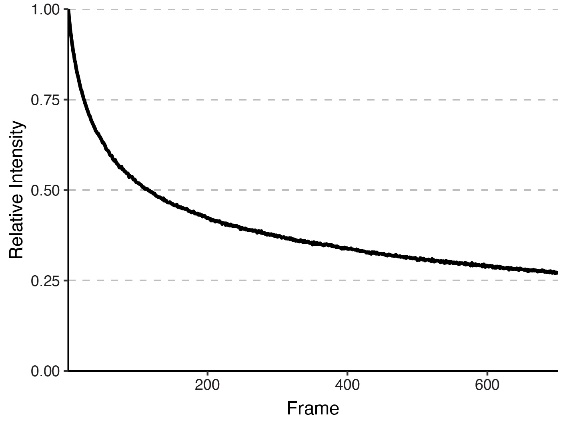

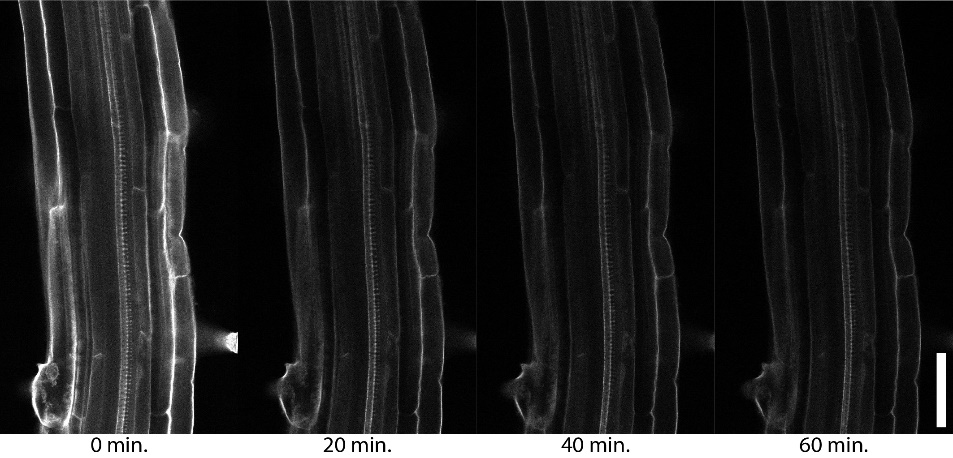


Fig S7: Confocal images of roots of Arabidopsis seedlings after fixation, clearing and staining with PyPe over 1h. Scale bar represents 100 µm. Excitation 405 nm, emission 425-525 nm.


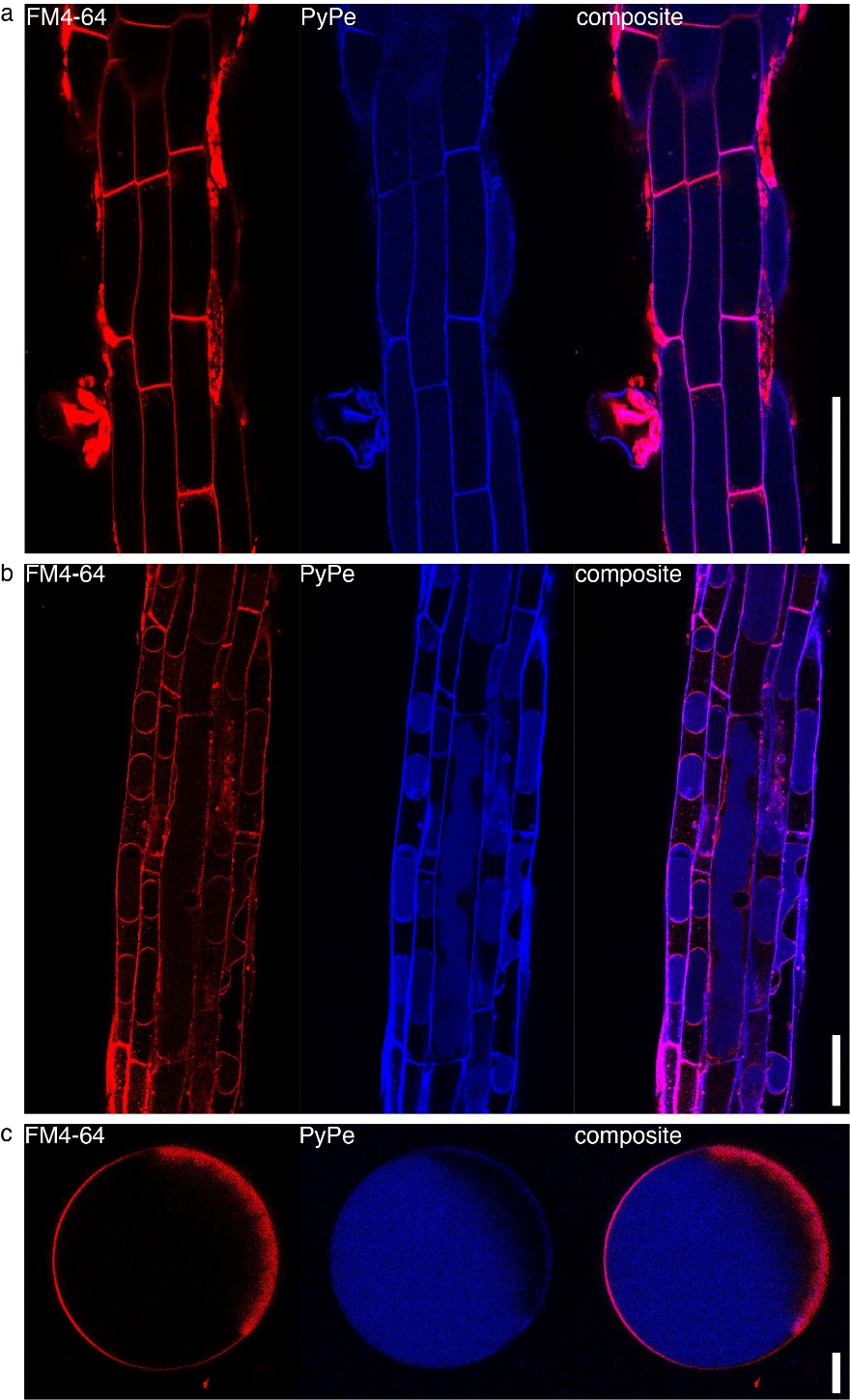


Fig S8: Arabidopsis roots staining with FM4-64 (left), **PyPe** (middle), and superimposition (right)
